# The Dilemma of Balancing Anti-Tumor Necrosis Factor-Alpha (Anti-TNF-α) Biologics for Psoriatic Arthritis Control With the Risk of Severe Systemic Infection

**DOI:** 10.7759/cureus.60476

**Published:** 2024-05-17

**Authors:** Yi-Fan Mai, Zhen-Cheng Hwang, Yung-Tsai Lee, Hsiao-Yi Lin

**Affiliations:** 1 Division of Allergy, Immunology, and Rheumatology, Department of Internal Medicine, Shin Kong Wu Ho-Su Memorial Hospital, Taipei, TWN; 2 Department of Internal Medicine, Shin Kong Wu Ho-Su Memorial Hospital, Taipei, TWN; 3 Division of Cardiology, Heart Center, Cheng Hsin General Hospital, Taipei, TWN; 4 Clinical Research Center and Division of Allergy, Immunology and Rheumatology, Department of Medicine, Cheng Hsin General Hospital, Taipei, TWN

**Keywords:** tavr (transcatheter aortic valve replacement), hypopyon uveitis, mtx, anti-tnf-α, jak inhibitor, infective endocarditis, adalimumab, biologics, psoriatic arthritis

## Abstract

The treatment landscape for psoriatic arthritis (PsA) has evolved significantly with the introduction of biologic therapies, such as adalimumab, which effectively inhibits tumor necrosis factor-alpha (TNF-α) activity. However, despite their efficacy in controlling inflammation, biologic therapies are associated with heightened risks of infectious complications and malignancies. We present a case of a 66-year-old female with PsA treated with adalimumab who presented with recurrent systemic bacterial infections. Despite attempts to adjust dosing intervals to minimize infection risks, the patient experienced severe complications, including urosepsis, endocarditis, and liver abscesses. The dilemma arises in balancing PsA control with anti-TNFα therapy while minimizing infection risks. Current evidence supporting prophylactic antibiotics in such cases is limited, and determining the next steps for treatment involves challenging decisions such as withholding TNF inhibitors or switching to alternative immunomodulators. This case underscores the need for further research into prophylactic treatment and monitoring protocols to manage recurrent infections during anti-TNF-α therapy effectively.

## Introduction

The treatment landscape for psoriatic arthritis (PsA) has significantly progressed due to the rapid evolution of biologic therapies. Inhibiting tumor necrosis factor-alpha (TNFα) initiates a cascade that reduces the expression of other proinflammatory cytokines and diminishes the recruitment of activated cells, consequently mitigating inflammation in both joints and skin [1]. Adalimumab, a recombinant human IgG1 monoclonal antibody (mAb), specifically targets and inhibits human TNF-α activity. It is Food and Drug Administration (FDA)-approved for various autoimmune diseases, including plaque psoriasis, PsA, rheumatoid arthritis, ankylosing spondylitis, and Crohn's disease in the United States.

While biologic therapies have demonstrated prominent efficacy in disrupting the inflammation process, there is also a reported elevated risk of infectious complications or even malignancies [2], becoming a significant concern for both attending physicians and patients. Here, we present a case of a patient with psoriatic arthritis who developed a recurrent systemic bacterial infection, with the recent episode affecting multiple organs, including the eyes, heart, and liver, after receiving adalimumab. This case serves as a reminder of the risks associated with novel treatments and underscores the importance of implementing a comprehensive prophylactic and monitoring protocol for individuals receiving biologic therapies.

## Case presentation

A 66-year-old female, with a medical history of chronic hepatitis B infection and diagnosed with psoriatic arthritis at age 55, presented to our emergency department with nausea, general malaise, and chills persisting for 3 days. Her initial presentation of psoriatic arthritis included generalized scaling skin plaques involving the scalp, forehead, anterior and posterior trunk, and all limbs, along with sausage-like swelling over bilateral toes. Over the past four years, she has been receiving a bi-weekly dosage of 40 mg subcutaneous adalimumab to control psoriatic arthritis, as methotrexate appeared to be insufficient for disease control. Notably, within the first and second years of initiating adalimumab treatment, she experienced two episodes of severe urinary tract infection caused by Escherichia coli, both complicated with bacteremia.

**Figure 1:**
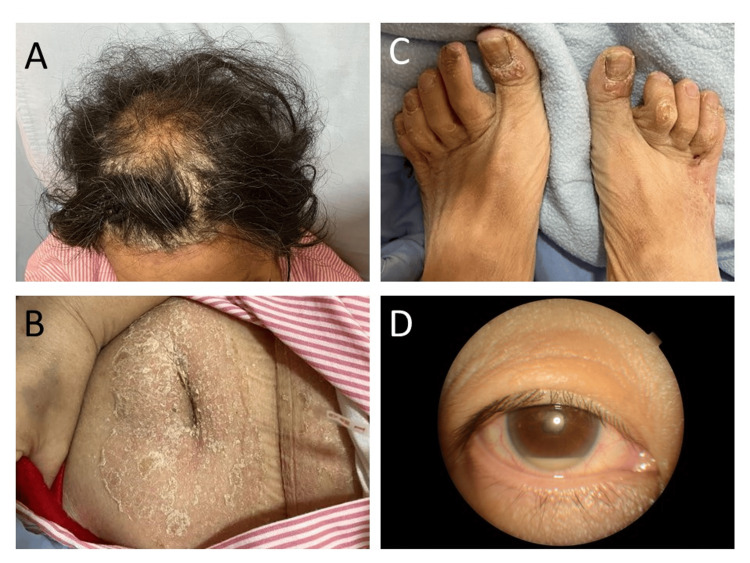
(A-C) Characteristic features of psoriasis patient presentation, (D) Anterior hypopyon uveitis (A) Diffuse scaly plaques over the scalp, (B) Diffuse scaly plaques over the trunk, (C) Proximal interphalangeal joint deformities of toes, (D) Redness over the right eye with anterior hypopyon uveitis, indicative of potential complications such as endophthalmitis

Upon triage at the emergency department, she was febrile with a body temperature of 39.0°C, a heart rate of 113 beats per minute, a blood pressure of 109/61 mmHg, a respiratory rate of 18 breaths per minute, and an oxygen saturation of 97% on ambient air. On physical examination, the patient exhibited skin plaques with scaling on the scalp, main trunk, and all four limbs, with prominent proximal interphalangeal joint (PIPJ) deformities on all limbs (Figures 1A-1C). No mucocutaneous ulcers were noted. Heart auscultation revealed a high-pitched, crescendo-decrescendo systolic ejection murmur heard over the right upper sternal border. The lungs were clear on auscultation. There was redness over the right eye and acute anterior hypopyon uveitis was confirmed through slit lamp evaluation by an ophthalmologist (Figure 1D).

**Figure 2:**
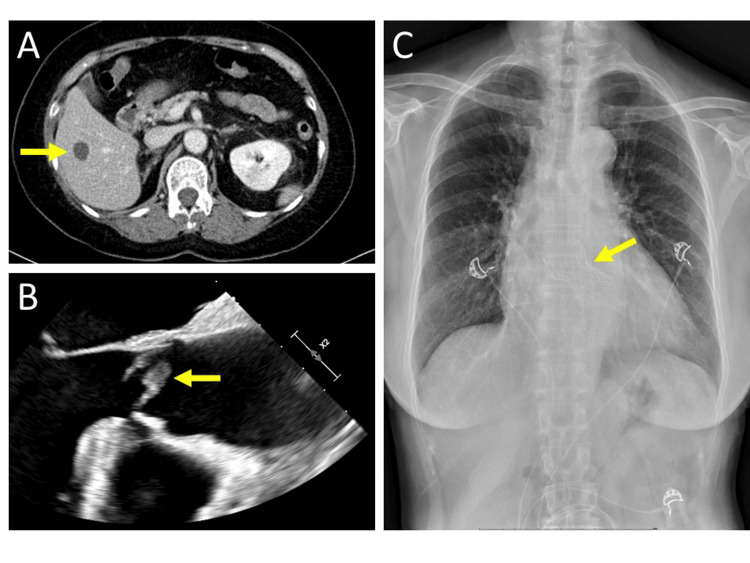
Severe infection characteristics associated with adalimumab and post-treatment follow-up (A) Abdominal contrast-enhanced CT scan showing a hypodense liver lesion (indicated by the yellow arrow), suggestive of a liver abscess. (B) Echocardiography image displaying a bicuspid aortic valve with vegetation attached (indicated by the yellow arrow). (C) Chest X-ray after transcatheter aortic valve replacement (TAVR) demonstrating the presence of a 34 mm CoreValve (indicated by the yellow arrow).

Laboratory testing revealed an elevated white cell count of 15,500 per microliter (reference range: 4000 to 11,000), with a differential count showing segmented neutrophils at 83.5% (reference range: 37 to 75) and bands at 10.0% (reference range: 0 to 2). Serum C-reactive protein demonstrated a significant elevation at 26.87 mg/dL (reference range: < 0.3 mg/dL). Other laboratory test results are shown in Table 1. For tuberculosis screening, Quantiferon-TB Gold was evaluated and yielded a negative result. Chest X-rays revealed clear lung fields and no cardiac enlargement. An abdominal contrast-enhanced CT scan demonstrated multiple small hypodense lesions over the liver, suggesting possible micro-abscesses (Figure 2A). Subsequent blood cultures yielded Staphylococcus aureus (SA) in two sets of culture collections, prompting admission for intravenous antibiotic treatment.

**Table 1:** Laboratory data assessment at triage

Variable	At triage, This hospital	Reference range, Adults, This hospital
Hemoglobin (g/dL)	13.9	11.0-16.0
White-cell count (*μL*)	15,500	4,000-11,000
Differential count (%)		
Segmented Neutrophils	83.5	37-75
Lymphocytes	3	20-50
Monocytes	2	4-10
Eosinophils	1.5	0-5
Basophils	0	0-2
Band	10	0-2
Platelet count (*μL*)	167,000	150,000-400,000
Sodium (mmol/L)	137	135-145
Potassium (mmol/L)	4.7	3.5-5.0
Creatinine (mg/dL)	1.24	0.65-1.10
Aspartate aminotransferase (IU/L)	116	8-38
C-reactive protein (mg/L)	26.87	<0.3
N-terminal pro-B-type natriuretic peptide (pg/mL)	2504	0-125
Troponin I (ng/mL)	0.762	0.00-0.04

The patient was administered intravenous Ceftriaxone and Daptomycin antibiotic treatment upon admission. However, progressive dyspnea was observed after hospitalization. Given the clinical suspicion of infective endocarditis, which was based on persistent dyspnea, the presence of a heart murmur, and positive blood cultures from Staphylococcus aureus (SA), initial transthoracic echocardiography (TTE) was conducted. TTE revealed the absence of vegetation, a preserved left ventricle ejection fraction (LVEF) of 65%, and severe aortic regurgitation with moderate aortic stenosis (aortic valve area = 1.35 cm^2^, mean pressure gradient = 31 mmHg). Despite the TTE findings, due to the high clinical suspicion of infective endocarditis, transesophageal echocardiography (TEE) was subsequently performed, revealing a bicuspid aortic valve with a 1.8 cm vegetation over the aortic valve (Figure 2B).

A multidisciplinary meeting involving a rheumatologist, cardiologist, and cardiovascular surgeon was convened for assessment and therapeutic decision-making. Following the 2021 European Society of Cardiology (ESC)/European Association for Cardiothoracic Surgery (EACTS) guidelines, a consensus was reached, emphasizing the primary surgical approach of eradicating the infected valvular tissue, followed by valve repair or replacement. However, the patient and her family expressed hesitation regarding the surgery, leading to its postponement. During hospitalization, follow-up blood cultures revealed no bacterial growth. After completing a total of six weeks of intravenous antibiotic treatment, imaging examinations, including a follow-up transthoracic echocardiogram (TTE), demonstrated the resolution of vegetation over the aortic valve. Subsequent contrast-enhanced abdominal CT indicated a complete diminishment of multiple liver micro-abscesses. The patient was then discharged.

However, six weeks later, she was readmitted due to acute decompensated heart failure (NYHA functional class III), complicated by acute pulmonary edema (Figure 2C), necessitating diuretics with inotropic agents. Given her advanced age and the high predicted mortality rate assessed by the European System for Cardiac Operative Risk Evaluation (EuroSCORE) II scoring system, transcatheter aortic valve replacement (TAVR) was favored over conventional valve repair surgery after shared decision-making with the patient and her family. TAVR with a 34 mm CoreValve (Medtronic plc, Minneapolis, Minnesota, US) was successfully performed (Figure 2D), resulting in the resolution of symptoms. The patient was discharged one week after TAVR and continues to follow up at the cardiology and rheumatology outpatient department.

## Discussion

TNF-α, an inflammatory cytokine primarily produced by activated macrophages and monocytes during acute inflammation, acts as a key regulator. It influences the expression of other pro-inflammatory cytokines such as interleukin-1 (IL-1) and interleukin-6 (IL-6) and plays a crucial role in orchestrating diverse signaling events within cells [3]. This mechanism can lead to necrosis or apoptosis, underscoring its importance in combating infections and cancers. Additionally, it is essential for the formation and maintenance of granulomas, serving as a critical component in suppressing tuberculosis (TB) [4]. TNF-α also enhances immune responses against viral pathogens such as hepatitis B virus (HBV) and varicella-zoster virus (VZV) [5]. Therefore, while TNF-α becomes a key target for therapeutic intervention in a redundant cytokine environment, improper inhibition may result in an increased risk of new TB infection, reactivation of latent TB, predisposition to opportunistic infections such as candida or aspergillus, or even a flare-up of viral infections [6]. Some reports have also linked anti-granulocyte antibody-related neutropenia to exposure to anti-TNF agents [7].

Currently, there are five TNF-α neutralization therapies approved by the FDA in the United States. These include the use of a soluble TNF-receptor fusion protein (etanercept) and monoclonal antibody-based drugs (infliximab, adalimumab, certolizumab, and golimumab) [8]. To mitigate potential infection-related adverse effects after using these novel medications, several guidelines and health authorities advise thorough monitoring for latent and active TB, HBV, human immunodeficiency virus (HIV), and VZV before initiating anti-TNFα biologics treatment [9-11]. TB screening should involve chest X-rays and an interferon-gamma release assay (IGRA), such as QuantiFERON^®^-TB Gold Plus (QFT^®^-Plus), instead of a tuberculin skin test (TST) as possible to prevent false positives from Bacille Calmette-Guerin (BCG) vaccination [10]. In cases of suspected TB infection, whether active or latent, initiate anti-TB medications before anti-TNFα biologics. For HBsAg-positive patients starting anti-TNFα treatment, antiviral therapy is advised. Before initiating anti-TNFα therapy, it is highly recommended to follow national health authority instructions for pneumococcal and seasonal influenza vaccinations. Additionally, consider HBV vaccination for seronegative patients and contemplate VZV vaccination for those without a prior infection [9].

In this case, a patient diagnosed with psoriatic arthritis, who was under adalimumab treatment, has been prescribed tenofovir (Vemlidy^®^) at a dosage of 300 mg once daily since the initiation of anti-TNFα biologics. Close surveillance of HBV activity and liver function was maintained. However, rather than experiencing an HBV flare-up, the patient encountered three episodes of systemic bacterial infections during the adalimumab treatment period, and the third episode led to severe comorbidity involving multiple organs, such as the eyes, heart, and liver, resulting in substantial medical costs, including those associated with TAVR. We attempted to extend the duration between adalimumab administrations from bi-weekly to monthly after the second episode of urosepsis. However, the patient experienced worsening psoriatic arthritis, characterized by bilateral toe pain, swelling, and stiffness, along with skin flakes, particularly on the main trunk. Consequently, we adjusted back to a bi-weekly frequency and carefully monitored urine routine during every outpatient visit to prevent recurrent urosepsis. Unfortunately, the patient experienced another episode of bacteremia, though the infection did not seem to be of urinary origin.

This complex scenario presents a dilemma in balancing the management of psoriatic arthritis with anti-TNFα therapy and mitigating the risk of bacterial infection. Bacterial components, such as lipopolysaccharides, stimulate the release of inflammatory mediators like TNF-α, IL-1, and IL-6, exacerbating disease progression. TNF-α, released early after infection, initiates a cascade of inflammatory responses, exacerbating inflammation and worsening the condition [12]. However, suppressing this pathway could potentially lead to uncontrolled infection [13]. Limited research exists on prophylactic antibiotics in patients undergoing prolonged anti-TNFα therapy. Existing literature typically extends beyond common pathogens like Staphylococcus aureus or Escherichia coli, instead emphasizing intracellular bacterial pathogens, such as Listeria, Legionella, and Salmonella, along with opportunistic infections like Pneumocystis jirovecii [2]. Despite the availability of prophylaxis recommendations, consensus on their use remains uncertain.

Furthermore, determining the next steps in her psoriatic arthritis treatment presents a challenging issue. This could involve either withholding the TNF inhibitor, switching to conventional disease-modifying antirheumatic drugs (DMARDs), or considering alternative novel biologics, such as Janus kinase (JAK) inhibitors, which recent studies have suggested may be superior to anti-TNFα therapy [14]. The decision will depend on shared decision-making with the patient, considering the risk-benefit analysis until further study or guidelines establish a formal protocol to address this situation.

## Conclusions

In conclusion, the intricate interplay between managing psoriatic arthritis with anti-TNFα therapy and averting bacterial infections poses a significant dilemma. Determining the optimal course for treating psoriatic arthritis in the presence of recurrent systemic bacterial infection presents a formidable challenge. The absence of a consensus on prophylactic antibiotics further complicates decision-making. Options such as withholding TNF inhibitors, transitioning to conventional DMARDs, or exploring alternative biologics like JAK inhibitors, based on shared decision-making with patients and considering the nuanced balance of benefits and risks, remain pivotal until formal guidelines or studies establish a standardized approach.
